# Response of a Hypersonic Boundary Layer to Freestream Pulse Acoustic Disturbance

**DOI:** 10.1155/2014/748504

**Published:** 2014-03-06

**Authors:** Zhenqing Wang, Xiaojun Tang, Hongqing Lv

**Affiliations:** College of Aerospace and Civil Engineering, Harbin Engineering University, Harbin 150001, China

## Abstract

The response of hypersonic boundary layer over a blunt wedge to freestream pulse acoustic disturbance was investigated. The stability characteristics of boundary layer for freestream pulse wave and continuous wave were analyzed comparatively. Results show that freestream pulse disturbance changes the thermal conductivity characteristics of boundary layer. For pulse wave, the number of main disturbance clusters decreases and the frequency band narrows along streamwise. There are competition and disturbance energy transfer among different modes in boundary layer. The dominant mode of boundary layer has an inhibitory action on other modes. Under continuous wave, the disturbance modes are mainly distributed near fundamental and harmonic frequencies, while under pulse wave, the disturbance modes are widely distributed in different modes. For both pulse and continuous waves, most of disturbance modes slide into a lower-growth or decay state in downstream, which is tending towards stability. The amplitude of disturbance modes in boundary layer under continuous wave is considerably larger than pulse wave. The growth rate for the former is also considerably larger than the later the disturbance modes with higher growth are mainly distributed near fundamental and harmonic frequencies for the former, while the disturbance modes are widely distributed in different frequencies for the latter.

## 1. Introduction

The accurate predictions about the aerodynamic, drag, and heat transfer rate of hypersonic vehicle surface can provide important basis to develop more efficient hypersonic vehicle in aerodynamic, thermal protection, and flight control designs, which is a challenge for hypersonic vehicle development [1]. These aerothermodynamics parameters depend considerably on boundary layer flow characteristics [2]. Therefore, investigations on boundary layer stability characteristics have very practical significance. Boundary layer is the regions near wall surface, where viscous force plays a critical role. Under certain circumstances, boundary layer flow can move from an orderly laminar state to a disorderly turbulent state, which is called transition. Aerodynamic frictional forces and heat transfer rate of hypersonic vehicle surface become elevated significantly once boundary layer flow changes from laminar to turbulent. Turbulence can make the friction drag on vehicle surface ten times larger. Therefore, the accurate predictions about the flow state of hypersonic vehicle surface have implications for new hypersonic vehicle development. In recent years, hypersonic boundary layer stability has caused wide public concern. A series of experimental and numerical investigations on the subject are performed [3–7], and a variety of theory interpretations and experiment analysis were conducted on the influential factor of boundary layer stability.

The process of boundary layer flow moving from laminar state to turbulent state is affected by many factors, such as freestream instability, wall surface roughness, wall temperature, and Reynolds number. Though the stability characteristics of hypersonic boundary layer had been investigated by many scholars, the transition mechanism of boundary layer, especially hypersonic boundary layer, is still not fully understood. The accurate prediction methods about transition position are still not fully reliable [8]. The hypersonic boundary layer stability characteristic is rather different from the boundary layer stability characteristic of incompressible flow, subsonic flow, and low Mach number supersonic flow. Under hypersonic condition, many new complex problems occur [9], which do not appear for low velocity flow conditions and should be understood, for instance, the appearance of second unstable disturbance mode, the effects of wall temperature on boundary layer stability, the sensitivity of flow factors to Mach number, and so forth. Thus, hypersonic boundary layer was not wholly explicable in terms of subsonic or low Mach number supersonic flow. The emergence of such problems makes the accurate transition prediction more difficult. For incompressible flow, subsonic flow, and low Mach number supersonic flow, boundary layer only contained unstable vorticity wave with low frequency, which is called the first disturbance mode or Tollmien-Schlichting (T-S) wave [10]. However, as the Mach number increases, the disturbance wave with high frequency occurs; apart from unstable vorticity wave with low frequency, there are a series of acoustic waves in boundary layer, which is unstable Mack2 mode. Mack2 mode becomes the least stable mode when Mach number is large enough [11]. Wang et al. [1] numerically studied the response of a Mach 8 flow over a 5.3° half-angle sharp wedge to wall blowing-suction and investigated the spatial development of boundary layer waves. They found that mode F, mode S, acoustic waves, and entropy/vorticity waves are simultaneously excited by wall blowing-suction. Maslov et al. [12] investigated the stability of a hypersonic shock layer on a flat plate. A new experimental technique is introduced for the investigation of artificially generated disturbances in planar laminar hypersonic boundary layers in [13]. Jiang et al. investigated [14] the instability wave propagation in boundary layer flows at subsonic through hypersonic Mach numbers, and three separate flow configurations are investigated. The linear and nonlinear developments of instability waves in a range of boundary layer flows are discussed. Fedorov and Khokhlov [15] investigated the prehistory of instability in a hypersonic boundary layer and presented a detailed analysis about how the forcing environmental disturbances enter into boundary layer and produce unstable wave that further develops and induces typical unstable wave in boundary layer. Based on direct numerical simulation (DNS) and linear stability theory (LST) analysis, Liang et al. studied the effects of wall temperature on stabilities of hypersonic boundary layer over a 7° half-cone-angle blunt cone under freestream small disturbance in [8] and found that the growth of disturbance waves is significantly affected by wall temperature; cooling the surface can accelerate unstable Mack II mode waves and decelerate Tollmien-Schlichting mode. As shown in previous studies, many investigations on the stability characteristic of hypersonic boundary layer have been presented, and most of these researches focused on the receptivity to freestream disturbance wave, response of hypersonic boundary layer to wall blowing-suction, the development of disturbance wave in boundary layer, and the effects of some flow parameters on boundary layer stability as well as laminar-turbulent transition. However, very few works were conducted on the effects of freestream pulse wave on hypersonic flow and boundary layer stability characteristic, whereas the interactions between freestream pulse wave and hypersonic flow as well as boundary layer are rather different from freestream continuous wave. Thus, the evolution mechanism of boundary layer disturbance wave and stability characteristic for the action of freestream continuous disturbance is rather different from pulse wave. Investigations on the area will help to understand the stability characteristic of hypersonic boundary layer under freestream pulse wave, which also can provide a different perspective for investigations on the stability of hypersonic boundary layer and is helpful to elucidate the underlying mechanisms of hypersonic boundary layer laminar-turbulent transition. Therefore, investigations on the receptivity and boundary layer stability for freestream pulse wave have very practical significance, and it is necessary to complete more systematic investigations.

In the present paper, hypersonic unsteady flows over a blunt wedge under the action of freestream pulse/continuous disturbance wave are computed. The interactions between freestream disturbance wave and hypersonic flow as well as boundary layer are analyzed. The response of hypersonic boundary layer to freestream disturbance wave and the evolution of boundary layer disturbance wave are investigated. The stability characteristics of hypersonic boundary layer over an 8° half-wedge-angle blunt wedge for freestream pulse wave and continuous wave were compared.

## 2. Governing Equations and Numerical Methods

### 2.1. Governing Equations

For steady and unsteady hypersonic flow computation, the Navier-Stokes equations in conservation form at Cartesian coordinates (*x*, *y*) are transformed into equations at general curvilinear coordinates (*ψ*, *ϕ*) and can be expressed as follows:

(1) $$\begin{array}{c} \frac{\partial \left(J^{-1}U\right)}{\partial t}+\frac{\partial F^{*}}{\partial \psi }+\frac{\partial G^{*}}{\partial \phi }+\frac{\partial F_{v}^{*}}{\partial \psi }+\frac{\partial G_{v}^{*}}{\partial \phi }=0, \end{array}$$

where the variables **U**, **J**, *t* are the state vector, the Jacobin matrix, and time, respectively, F* and G* are nonviscid terms at general curvilinear coordinates, and **F**_v_* and **G**_v_* are viscid terms at general curvilinear coordinates, while

(2) $$\begin{array}{c} F^{*}=\frac{F\psi_{x}+G\psi_{y}+U\psi_{t}}{J},\\ G^{*}=\frac{F\phi_{x}+G\phi_{y}+U\phi_{t}}{J},\\ F_{v}^{*}=\frac{F_{v}\psi_{x}+G_{v}\psi_{y}}{J},\\ G_{v}^{*}=\frac{F_{v}\phi_{x}+G_{v}\phi_{y}}{J}, \end{array}$$

where the variables **F** and **G** are nonviscid terms at Cartesian coordinates and **F**_*v*_ and **G**_*v*_ are viscid terms at Cartesian coordinates.

### 2.2. Numerical Method

To accurately simulate hypersonic unsteady flow filed under disturbances wave in freestream, a high-order direct numerical simulation method will be established and is used to solve compressible Navier-Stokes equations. The splitting of convection terms is conducted by Steger-Warming splitting method [16], as is shown in (3). Since the high-order weighted essentially nonoscillatory (WENO) methods have been widely implemented in the DNS of compressible turbulent flows, show strong robustness and high resolution, and are able to keep the higher-order approximations in smooth regions and to eliminate or suppress the oscillatory behavior near the discontinuities [17], positive convection and negative convection are discretized with 5th order upwind WENO scheme [18], as shown in (4) and (5), respectively. Viscous terms are discretized with 6th order center difference scheme [19], as is shown in (6); third order Runge-Kutta TVD type method [20] is employed for time advancing. To avoid the fact that accuracy of interior grid node is polluted, the 5th order WENO weighted scheme is used in the boundary points of positive convection terms, negative convection terms, and viscous terms.

Steger-Warming splitting method for convection terms splitting can be expressed as follows:

(3) $$\begin{array}{c} W=W^{+}+W^{-}, \end{array}$$

where the variables *W* ^−^ and *W* ^+^ are positive convection terms and negative convection terms, respectively.

The positive and negative convection terms are discretized by the 5th upwind scheme and can be expressed as follows in (4) and (5), respectively:

(4) $$\begin{array}{c} W^{+\prime }=\frac{1}{\Delta }(\sum_{N=1}^{6}m_{N}W_{j+3-N}^{-}), \end{array}$$

(5) $$\begin{array}{c} W^{-\prime }=\frac{1}{\Delta }\left(\sum_{N=1}^{6}n_{N}W_{j+4-N}^{+\prime }\right), \end{array}$$

where Δ is the grid spacing, *W* ^+′^and *S* ^−′^ are the difference approximation of the derivative of *W* ^+^ and *W* ^−^, respectively, and *m*_*i*_ and *n*_*i*_ are weighting coefficients.

Center difference scheme is employed for viscous terms discretion and can be expressed as follows:

(6) $$\begin{array}{c} H^{\prime }=\frac{1}{\Delta }\left(\sum_{N=1}^{3}K_{N}\left(H_{j+N}-H_{j-N}\right)\right), \end{array}$$

where the variables *H*, *H*′, and *K*_*i*_ are viscous terms, the difference approximation of the derivative of viscous terms, and weighting coefficient, respectively.

**Figure 1:**
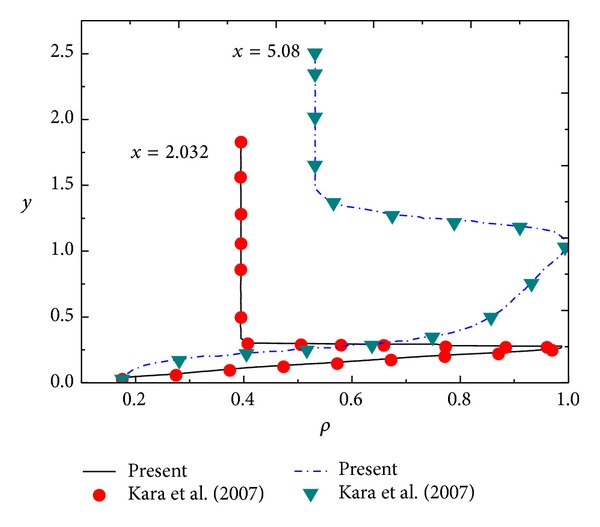
Comparison of density profile with results of Kara et al.

**Figure 2:**
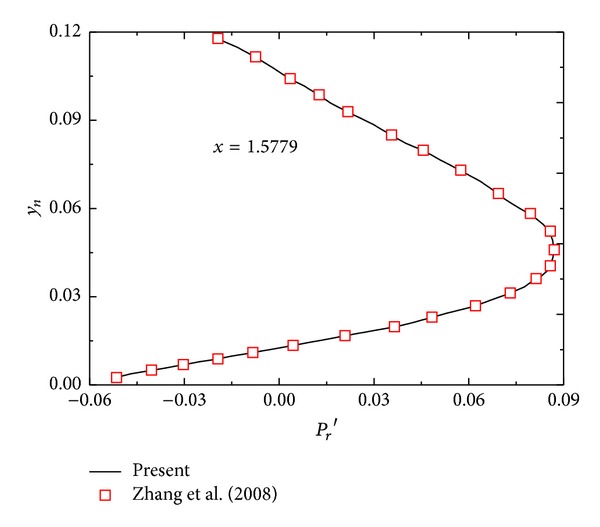
Comparison of the real part of Fourier transform for pressure disturbance with Zhang et al.'s result.

The numerical simulations of two similar modes are conducted as follows: (1) Mach number of 6 over a blunt wedge with a half-wedge-angle *θ* = 5° under freestream acoustic disturbances and (2) Mach number of 15 over a blunt body with *θ* = 5° under freestream acoustic disturbances, entropy disturbances, and vorticity disturbances. Figures 1 and 2 show the comparison of density profile with results of Kara et al. [17] and the comparison of the real part of Fourier transform for pressure disturbance with Zhang et al.'s result [19]. From Figures 1 and 2, it can be seen that the effectiveness of the numerical method is demonstrated.

## 3. Computational Conditions

**Figure 3:**
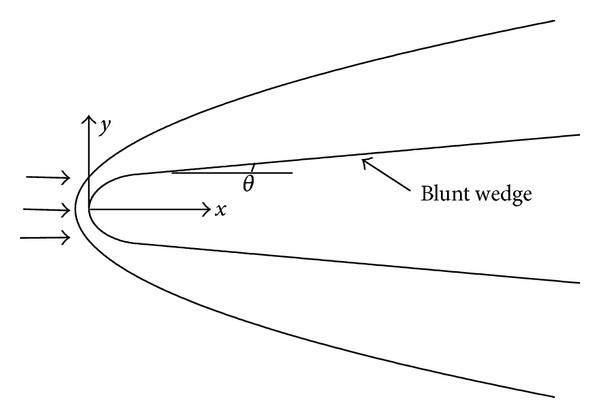
Computational mode.

Based on a hypersonic flow over a wedge with blunt noses, the numerical simulations of hypersonic unsteady flow under freestream fast acoustic wave are performed. The computational mode and schematic diagram are shown in Figure 3. Freestream condition and extrapolation are employed at computational field upstream and the exit of hypersonic flow fields, respectively. No-slip and isothermal wall conditions are enforced at the wall surface. The solutions presented in this paper are resolved by 300 × 120 grids. The mesh grid density in this paper matches that in Zhang et al.'s and Prakash's investigations [19, 21]. The stretching function is used to cluster more points in noses area and boundary layer. The effectiveness of the stretching method is demonstrated in Figures 1 and 2. An asymmetry condition is introduced at *y* = 0. The parameters used in the paper are dimensionless. The details are expressed as follows: the velocity, the length scales, the density *ρ*, the pressure *P*, the temperature *T*, and the time *t* are nondimensionalized by the freestream velocity, the nose radius *R*, the freestream density *ρ*_*∞*_,  *ρ*_*∞*_ *u*_*∞*_ *u*_*∞*_, the freestream temperature *T*_*∞*_, and *R*/*u*_*∞*_, respectively. Based on the dimensionless method above, nondimensional computation is conducted in this paper; the results are nondimensional. For example, the nondimensional frequency *f* is obtained by *u*_*∞*_/*R*. The Reynolds number, *Re*_*n*_ = *ρ*_*∞*_ *Ru*_*∞*_/*μ*_*∞*_, based on freestream parameters and the cone's nose radius, is equal to 10000, where *μ*_*∞*_ is viscosity coefficient. The wave fields are represented by disturbances of instantaneous flow variables with respect to the local steady base flow variables at the same location. Subscripts “*∞*” and “′” denote freestream condition and the disturbances of instantaneous flow variables, respectively. The flow parameters are shown in Table 1. The variables Re, *Ma*_*∞*_, *T*_*∞*_, *T*_*w*_, *α*_*n*_, and *θ* are Reynolds number, freestream Mach number, freestream temperature, wall temperature, angle of attack, and half-wedge-angle, respectively.

Freestream fast acoustic continuous and pulse waves are separately introduced to the upstream end of the steady flow field without disturbance to do direction numerical simulation of hypersonic unsteady flow at *t*1 = 52. The form of the disturbance wave employed can be expressed as follows:

(7) L={0t<t1LAei(kx−((F·Re)/106)t+(π/2))t1≤t<t20t≥t2,

where the matrix $L={\left[\begin{array}{cccc} u^{\prime } & v^{\prime } & p^{\prime } & \rho^{\prime } \end{array}\right]}^{T}$, $L_{A}={\left[\begin{array}{cccc} A & 0 & A/Ma & AMa \end{array}\right]}^{T}$, the variables *u*′,  *v*′,  *p*′, and *ρ*′ are the disturbance values of the velocity along *x*-axis, velocity along *y*-axis, pressure, and density, respectively, the amplitude *A* = 0.06, wave number *k* = 3.1446 × 10^−4^, generalized frequency *F* = 50 *π*, and freestream Mach *Ma* = 6.0. When continuous wave in freestream is simulated, *t*2 should be taken to infinity theoretically; however, *t*2 should only be large enough to enable the flow field to reach period state in practice. When pulse wave in freestream is simulated, the length of time is 2.0(*u*_*∞*_/*R*), namely, 1/2 cycle; *t*1 and *t*2 should be taken to 52.0 and 54.0, respectively.

## 4. Numerical Results and Discussion

### 4.1. Response of Hypersonic Flow Field to Pulse Fast Acoustic Wave

**Figure 4:**
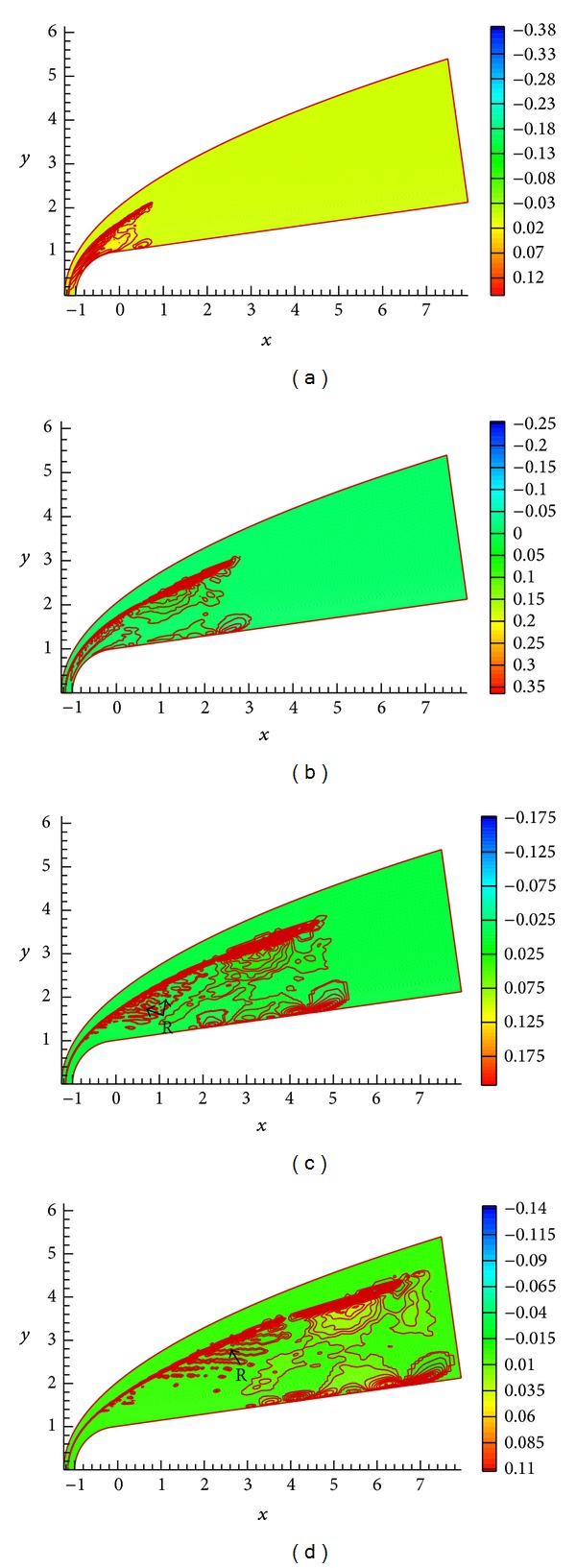
Contours of velocity disturbances along *y*-axis *v*′(*x*, *y*, *t*) at different times.

The contours of velocity disturbances along *y*-axis at *t* = 54.0, 56.0, 58.0, and 60.0 are shown in Figures 4(a), 4(b), 4(c), and 4(d), respectively. The instantaneous velocity disturbances along *y*-axis on the areas of interactions of disturbance wave with the bow shock and shock layer and inside the boundary layer are significantly affected. In particular, the flow velocity gradient near wall surface is larger. There exist significant differences between velocity disturbance modes outside and inside the boundary layer. The forcing disturbance wave interacts with bow shock and then enters the boundary layer; a part of disturbance wave propagated along streamwise, and the other repeat moved between shock and nose [8]. The reciprocating motion of disturbance wave is continual for freestream continuous wave, while the number of reflection is limited for freestream pulse wave due to viscous dissipation. It should be mentioned that the velocity disturbances behind bow shock still exist when pulse wave leaves away, as the mark R shown in Figure 4. This is the result of the residual reflection wave being enlarged by shock wave, and then the residual reflection wave will be dissipated due to viscous dissipation. Therefore, the residual reflection wave only appears near the bow shock. Disturbance wave with different modes will be generated after the forcing disturbance wave interacts with boundary layer; the generated waves further interact with boundary layer and induce more unstable modes [8]. The interactions between boundary layer and unstable wave become more complicated due to the reflection between bow shock wave and wall.

**Figure 5:**
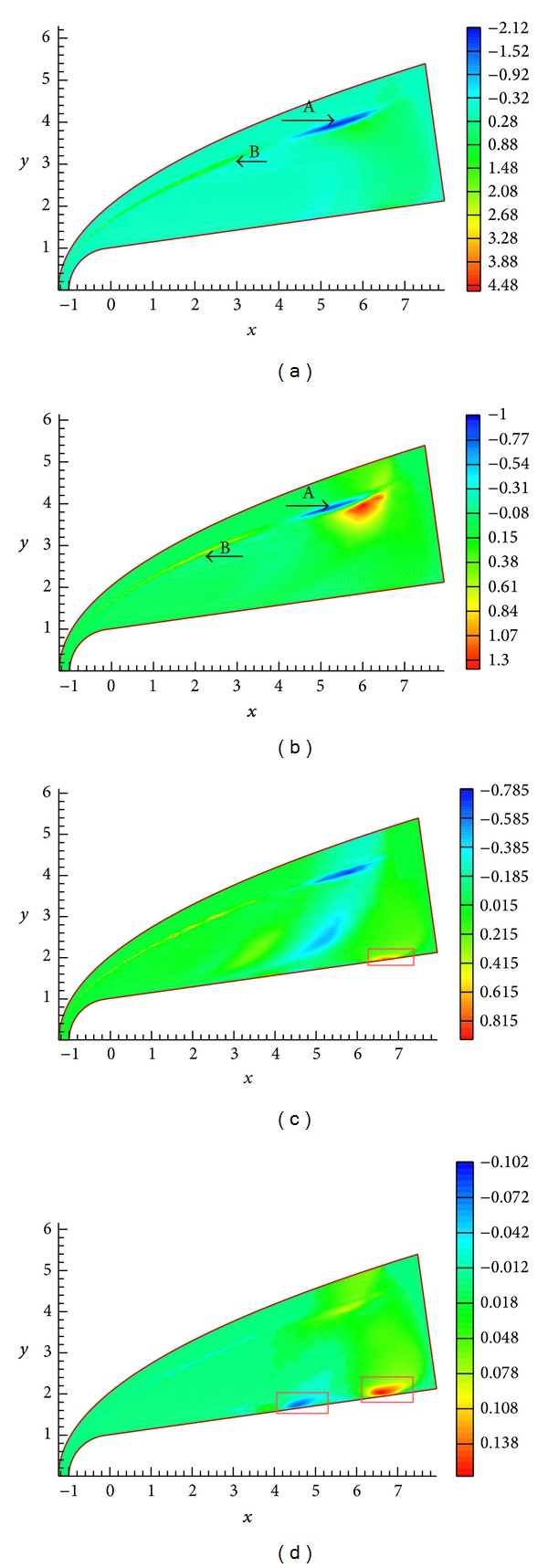
Contours of pressure *P*′(*x*, *y*, *t*), density *ρ*′(*x*, *y*, *t*), temperature *T*′(*x*, *y*, *t*), and velocity disturbance.

The contours of pressure *P*′(*x*, *y*, *t*), density *ρ*′(*x*, *y*, *t*), temperature *T*′(*x*, *y*, *t*), and velocity disturbance are shown in Figures 5(a), 5(b), 5(c), and 5(d), respectively. As seen, bow shock is bent obviously inwards when being subjected to pulse wave. The pressure *P*′(*x*, *y*, *t*), density *ρ*′(*x*, *y*, *t*), and temperature *T*′(*x*, *y*, *t*) become smaller sharply in the deformation area of shock wave, as the mark A shown in the figure. The amplitude of flow variables change is significantly larger than that of initial disturbance in freestream. As discussed above, a part of disturbance wave will be reflected between shock and nose [8]. It should be noted that the bow shock wave slightly deforms when being subjected to the refection wave, as the mark B shown in the figure. Liang et al. [8] studied the evolution of continuous small disturbance waves in hypersonic flow by using direct numerical simulation (DNS) and found that, because of the normal shock wave, the forcing disturbance in freestream is enlarged.  Figure 5 shows that the disturbance amplitude of flow variables is enlarged sharply relative to that of initial disturbance in freestream. The numerical results agree with Liang et al.'s results. From the figure, the velocity and temperature near the wall surface are changed sharply as confronts freestream acoustic disturbances, as the rectangular mark shown in the figure. Obviously, the structure of strong shear flow and the thermodynamics properties in boundary layer are changed significantly under freestream pulse wave; the structure of strong shear flow and the thermal state near wall surface will affect the behavior of hydrodynamic stability [2]. The evolvement of unstable disturbance waves in boundary layer and the boundary layer stability characteristic will be discussed in the following.

### 4.2. Evolvement of Unstable Disturbance Waves in Boundary Layer

**Figure 6:**
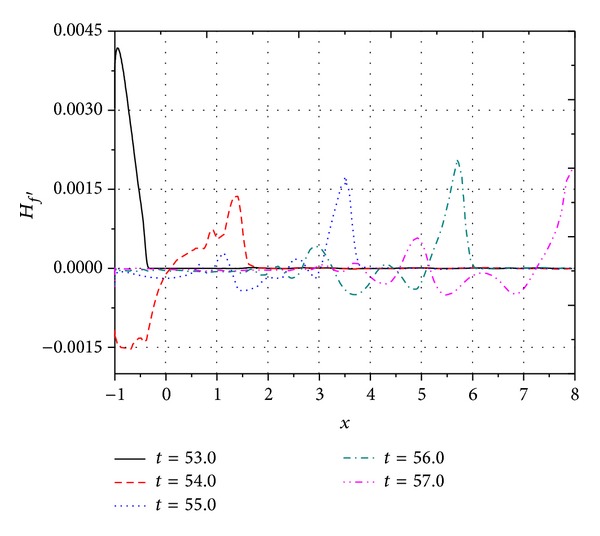
Distribution of wall heat flux disturbance *H*_*f*_′(*x*, *y*, *t*) at different times.

Figure 6 shows the variation of heat flux disturbance on the wall surface at different times (*t* = 53.0, 54.0, 55.0, 56.0, 57.0, 58.0, 59.0, 60.0, and 61.0). It shows that when the flow field was subjected to the semisinusoidal shape of the pulse disturbance wave, the heat flux disturbance firstly increases and then decreases, the trend of which is analogous to freestream disturbance wave. However, when the pulse disturbance wave passes through entirely, the effect remains. Furthermore, the disturbance amplitude will undergo a damped oscillation until the amplitude decreases to 0. It is due to the reflection of the disturbance wave between bow shock and wall. From Figure 6, it is clearly indicated that the heat flux on wall changed sharply under freestream pulse acoustic disturbance with *A* = 6 × 10^−2^. Since heat flux is a characteristic variable of heat conductivity in boundary layer, the thermal conductivity of boundary layer flow is significantly affected by freestream pulse wave. The influences of the thermal conduct mechanism of boundary layer flow on the evolution of disturbance wave modes and boundary layer stability state were confirmed in [2, 22, 23]. Meanwhile, thermal surface state will be changed by the thermal conduct on the wall surface. Hirschel [2] found that the thermal surface state has a strong back-coupling to the state of the boundary layer. It is reasonable to believe that the behaviors of hydrodynamic stability, even laminar-turbulent transition, will be affected under freestream pulse disturbance.

**Figure 7:**
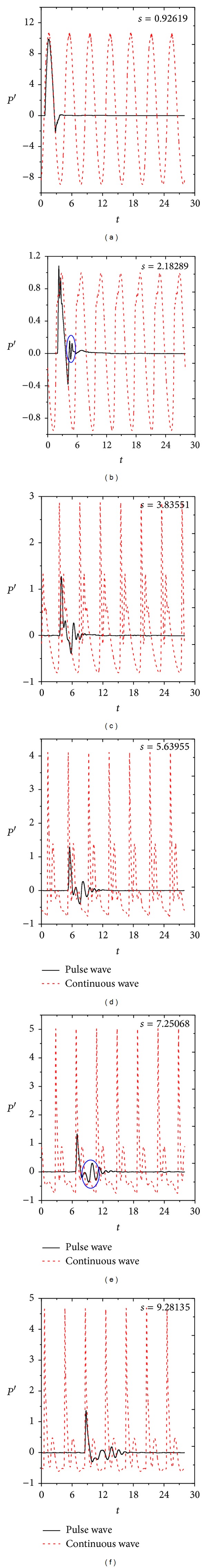
Pressure disturbance at different surface locations varying with time.

Before broaching the following discussions, it should be mentioned that a fitted coordinate system *s* is introduced to represent the distance from the points on the generatrix of blunt wedge to the stagnation point. The relationship between *x* and *s* is expressed in [8]. In order to study the disturbance wave evolvement in boundary layer, the comparison of variation of pressure disturbance locations with time between freestream continuous wave and pulse wave at different surface locations is given in Figure 7. It can be seen that when the flow field is subjected to freestream pulse disturbance wave, the pressure disturbance firstly increases and then decreases, the trend of which is similar to freestream pulse wave. However, when the pulse disturbance wave passes through entirely, the effect remains. The disturbance amplitude will undergo a damped oscillation until the amplitude decreases to 0. The damped oscillation is due to the reflection of disturbance waves between wall and bow shock, as the rounded mark shown in the figure. From the variation of pressure disturbance in periodic states under the action of freestream continuous wave as shown in Figure 7, the oscillation also can be seen. There exist the disturbance wave propagation and the complex mutual interferes of the reflection wave and boundary layer, which complicate the boundary layer flow. This leads to the fact that the pressure disturbance is changed from relatively unitary shape in upstream to complex shape in downstream. From Figure 7, for both pulse wave and continuous wave, the amplitudes of pressure disturbance are enlarged significantly relative to initial freestream disturbance. In general, due to the fact that the amplification caused by normal shock wave (approximates) in nose is larger than that caused by oblique shock wave in nonnose, the amplitude of pressure disturbance in nose is significantly larger than that in non-nose. This agrees with Liang et al.'s results [8]. It should be mentioned that, for continuous wave, the pressure disturbance is periodic; however, for pulse wave, the pressure disturbance changed with the time. That is, there only exists spatial evolution (along streetwise) in boundary layer for continuous wave, while there exist not only spatial evolution but also temporal evolution in boundary layer for pulse wave.

**Figure 8:**
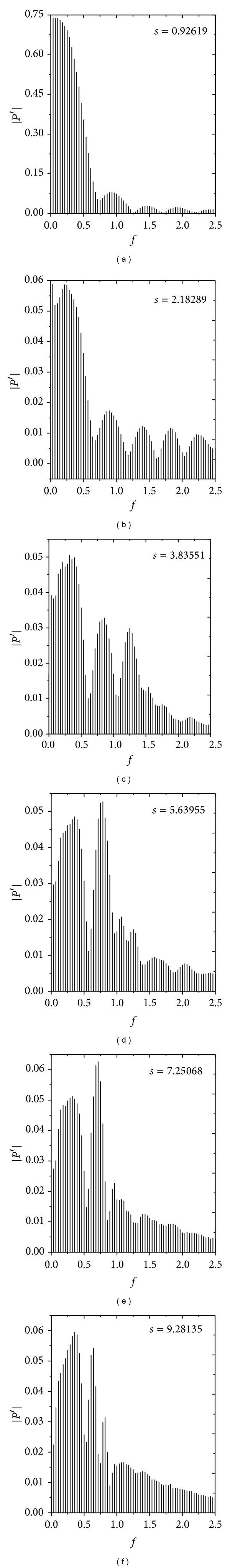
Fourier frequency analysis of pressure disturbance at different surface locations under freestream pulse fast acoustic wave.

The temporal signals of pressure disturbance are decomposed into frequency signals using Fourier transform.  Figure 8 shows the Fourier frequency analysis of pressure disturbance at different surface locations under freestream pulse fast acoustic wave. It can be seen that (a) the frequencies of most disturbance are less than 0.5 in nose boundary layer. There are 5 main disturbance mode clusters at *s* = 2.18289; however, the number is reduced from 5 at *s* = 2.18289 to 2 at *s* = 5.63955, and the two clusters are mainly distributed near *f* = 0.25 and *f* = 0.75. When 5.63955 < *s* < 7.25078, the amplitude of *f* = 0.75 is once larger than that of *f* = 0.25, and this makes the dominant mode transfer in boundary layer. It indicates that the development of dominant mode in boundary layer restrains the growth of other modes. (b) The Fourier amplitude of pressure disturbance at *s* = 0.92616  (*s* < *π*/2, the nose region) is greater than that at other locations. It is mainly owing to the bow wave going from normal shock on the nose region to oblique shock wave on the non-nose region. (c) With the development of disturbance waves in boundary layer along streamwise from upstream to downstream, various tends are revealed for the different frequency disturbance modes, such as continuous growth, decay, and first growth and then decay. It indicates that there is competition among different modes in boundary layer. In general, when *s* > *π*/2, the Fourier amplitude of pressure disturbance of low frequency mode (*f* < 0.25) decreases and the Fourier amplitude of high frequency (*f* > 0.5) increases. The ratio between low frequency component and high frequency component transformed quickly, and the high frequency components increase quickly. With the development of disturbance waves along streamwise from upstream to downstream, the frequency band narrows.

**Figure 9:**
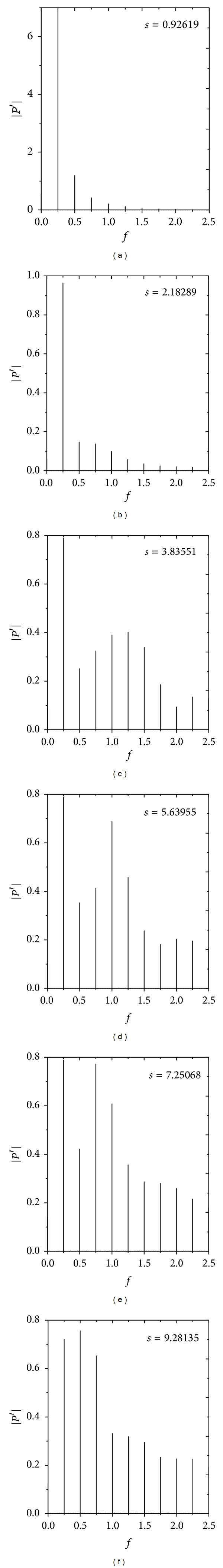
Fourier frequency analysis of pressure disturbance at different surface locations under freestream continuous fast acoustic wave.

Figure 9 shows the Fourier frequency analysis of pressure disturbance at different surface location under freestream continuous fast acoustic wave. It indicates that, under freestream continuous wave with single frequency, the disturbance modes in boundary layer are mainly distributed near fundamental frequency and harmonic frequencies (i.e., *P*_*n*_, where *n*  is positive integer), and the amplitudes of the other modes (*n* is not integer) are tiny. Comparing Figures 8 and 9, it can be seen that, (1) for both freestream continuous wave and pulse wave, the amplitudes of fundamental frequency (for freestream continuous wave) near fundamental frequency (freestream pulse) decrease sharply in the nose boundary layer. With the development of disturbance waves along streamwise from upstream to downstream, the components of high frequency disturbance modes (the frequency is larger than the second harmonic; that is, *f* > 0.5) increase quickly. (2) The Fourier amplitudes of pressure disturbance in boundary layer under freestream continuous wave are considerably larger than those under freestream pulse wave; there is an order of magnitude difference between the two conditions. Obviously, this is due to the fact that the disturbance energy in boundary layer under freestream continuous wave is persistent, while that under freestream pulse wave is temporary. (3) Under freestream continuous wave, the disturbance modes in boundary layer are mainly distributed near fundamental frequency and harmonic frequencies, and the amplitudes of the other modes are very tiny, while, under freestream pulse wave, the disturbance modes in boundary layer are widely distributed in different modes. Namely, the frequency band for the former is narrower than the latter. This means that the disturbance energy is transferred from both fundamental frequency and harmonic frequencies to other modes, which increases the amplitudes of other modes (*P*_*n*_, where *n* is not integer). Obviously, it has also become another important reason why the Fourier amplitudes of pressure disturbance under continuous wave are considerably larger than those under pulse wave. (4) For pulse wave, there are only 2 main disturbance mode clusters in boundary layer when *s* ≥ 5.63955. The two main mode clusters are distributed near fundamental frequency and the third harmonic frequency, and the other modes remain basically stable, even to attenuate. However, for continuous wave, there are still 3 main disturbance modes in boundary layer when *s* = 9.28315. The 3 main modes are fundamental frequency, the second harmonic frequency, and the third harmonic frequency.

**Figure 10:**
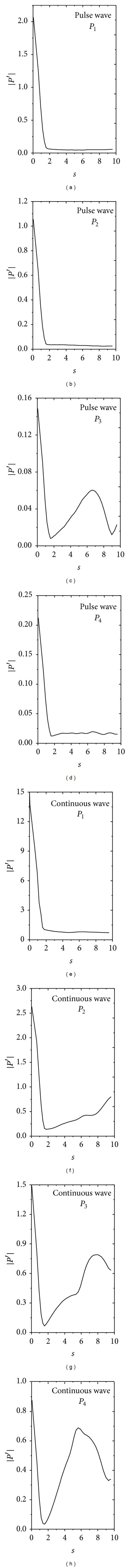
Comparison of pressure disturbances amplitudes in boundary layer for both pulse and continuous fast acoustic waves.

Figure 10 shows the comparison of pressure disturbances amplitudes for fundamental frequency (*P*_1_) and harmonic frequencies (*P*_2–4_) in boundary layer under both pulse wave and continuous wave. It can be seen that, for both pulse wave and continuous wave, the pressure disturbance amplitudes for fundamental frequency (*P*_1_) and harmonic frequencies (*P*_2–4_) in nose boundary layer are larger than those in the non-nose boundary layer; the amplitudes of pressure disturbance for fundamental frequency (*P*_1_) are significantly larger than those for harmonic frequencies (*P*_2–4_) in the nose boundary layer. It indicates that the nose boundary layer is dominated by fundamental frequency (for freestream continuous wave) or the modes near fundamental frequency (for freestream pulse wave). The results are consistent with investigations by Zhang et al. [19]. It also can be seen that, for both fundamental frequency and harmonic frequencies, the disturbance amplitudes for freestream pulse wave are significantly less than those for continuous wave. It indicates that the growth of boundary layer disturbance wave for pulse wave is much later than that for continuous wave. For both pulse wave and continuous wave, fundamental frequency remained basically stable in non-nose boundary layer. However, the variation of harmonic frequencies in non-nose boundary layer along streamwise for continuous wave differs from that for pulse wave. For the former, there exists a significant growth for the second harmonic frequency *P*_2_, the third harmonic frequency *P*_3_, and the fourth harmonic frequency *P*_4_, when *s* > *π*/2. For the latter, only the third harmonic frequency *P*_3_ increases significantly when *s* > *π*/2; the second harmonic frequency *P*_2_ and the fourth harmonic frequency *P*_4_ remain basically stable, even to attenuate when *s* > *π*/2.

**Figure 11:**
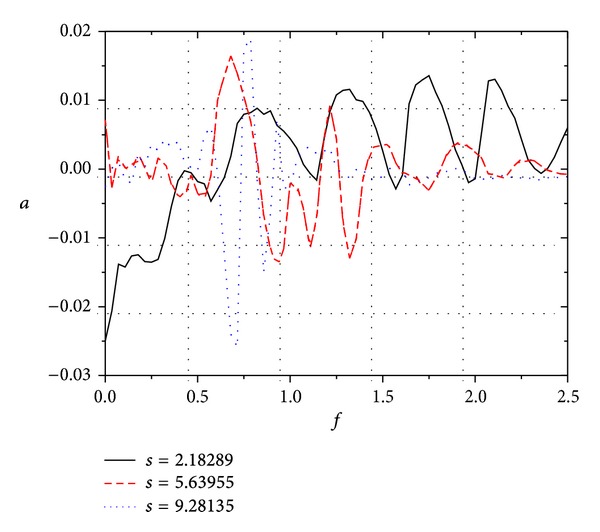
Growth of different frequency disturbances in boundary layer along streamwise under freestream pulse fast acoustic wave.

Figure 11 shows the growth rate *α* for different frequency disturbances in boundary layer along streamwise under freestream pulse wave. It can be seen that, when *s* = 2.18289, the growth of pressure disturbance mode with low frequency (*f* < 0.5) is negative, and the disturbance amplitudes tend to decline; the decay of low frequency modes becomes slow with the frequency increasing; the growth of pressure disturbance modes for *f* > 0.5 generally increases; there exist 4 disturbance mode clusters with high growth and 4 growth peaks. When *s* = 5.63955, the decay of pressure disturbance modes for *f* < 0.25 becomes slow, even to grow; the pressure disturbance modes for 0.5 < *f* < 0.8 continue to grow. When *f* > 0.8, the growth of different frequency disturbances in boundary layer is relatively larger at *s* = 2.18289, while the value becomes small, even to decay. When *s* = 9.28135, the amplitude of growth rate for *f* > 1.0 and *f* < 0.5 is tiny, which maintains total stability; the modes for 0.7 < *f* < 0.8, namely, the third harmonic frequency, increase sharply; these indicate that the development of dominant mode inhibited the other modes in boundary layer.

**Figure 12:**
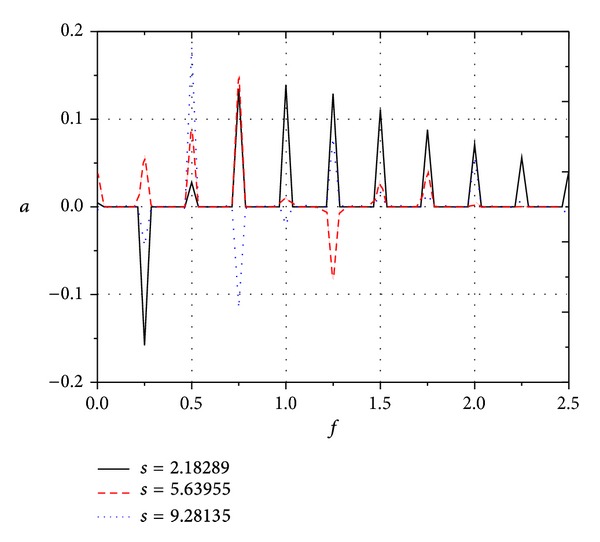
Growth of different frequency disturbances in boundary layer along streamwise under freestream continuous fast acoustic wave.

Figure 12 shows the growth for different frequency disturbances in boundary layer along streamwise under freestream continuous wave. It shows that the growth rate of the disturbance modes for fundamental frequency and harmonic frequencies (i.e., *P*_*n*_, where  *n* is positive integer) is significantly larger than for other modes (*n* is not integer). When *s* = 2.18289, the fundamental frequency decays and all harmonic frequencies increase. When *s* = 5.63955, the fundamental frequency increases slowly; all harmonic frequencies increase except the disturbance modes for *f* = 1.25 (*P*_5_). When *s* = 9.28135, *P*_2_ continued to grow, *P*_3_ began to decay, and the other modes slide into a lower-growth or no-growth state, which presents a total stability. From Figures 11 and 12, (1) for both pulse wave and continuous wave, with the development of disturbance waves in boundary layer from upstream to downstream, most of disturbance modes slide into a lower-growth or decay state, which is tending towards stability. (2) The amplitudes of the growth rate of disturbance modes in boundary layer under freestream continuous wave are considerably larger than those under freestream pulse wave. (3) The disturbance modes with higher growth are mainly distributed near fundamental frequency and harmonic frequencies under freestream continuous wave, while the disturbance modes with higher growth are widely distributed in different modes under freestream pulse wave.

## 5. Conclusions

The response of hypersonic boundary layer to freestream pulse acoustic wave is analyzed, and the stability characteristics of boundary layer under freestream pulse and continuous fast acoustic wave are compared. We draw some conclusions.

1. Under freestream pulse acoustic disturbance, the bow shock is bent obviously, and the thermal conductivity of strong shear flow in boundary layer is significantly changed. Various tends are revealed for the different frequency disturbance modes along streamwise, such as continuous growth, decay, and first growth and then decay. With the development of disturbance waves along streamwise, the number of main disturbance mode clusters reduced quickly, and the frequency band narrows. The dominant mode in boundary layer restrains the growth of other modes. There is competition among different modes in boundary layer. There also is disturbance energy transfer between fundamental frequency and harmonic frequencies (*P*_*n*_, where  *n* is positive integer) and other modes (*n* is not integer).
2. For both pulse wave and continuous wave, the nose boundary layer is dominated by fundamental frequency or the modes near fundamental frequency. The amplitudes of fundamental frequency or near fundamental frequency decrease sharply in the nose boundary layer. However, the fundamental frequency remains basically stable, and the components of high frequency disturbance modes (*f* > 0.5) increase quickly along streamwise in non-nose boundary layer. The Fourier amplitudes of disturbance in boundary layer under freestream continuous wave are considerably larger than those under freestream pulse wave; there is an order of magnitude difference between the two conditions. The disturbance modes in boundary layer are mainly distributed near fundamental frequency and harmonic frequencies, and the amplitudes of the other modes are tiny under freestream continuous wave, while the disturbance modes in boundary layer are widely distributed in different modes under freestream pulse wave. Namely, the frequency band for the former is narrower than the latter.
3. For both pulse wave and continuous wave, with the development of disturbance waves along streamwise, most of disturbance modes slide into a lower-growth or decay state, which is tending towards stability. The amplitudes of the growth rate of disturbance modes in boundary layer under freestream continuous wave are considerably larger than those under freestream pulse wave. The disturbance modes with higher growth are mainly distributed near fundamental frequency and harmonic frequencies for the former, while the disturbance modes with higher growth are widely distributed in different modes for the latter.

## Figures and Tables

**Table 1:** Computational conditions.

Re	Ma_∞_	*T*_*∞*_ (K)	*T*_*w*_ (K)	*α*_*n*_ (°)	*R* (mm)	*θ* (°)
10000	6	169	200	0	1	8
